# Evaluation of Palatal Thickness for the Placement of MARPE Device among a Cohort of Iraqi-Kurdish Population: A Retrospective CBCT Study

**DOI:** 10.1155/2024/6741187

**Published:** 2024-08-28

**Authors:** Fedil Andraws Yalda, Omar Fawzi Chawshli, Shaho Ziyad Al-Talabani, Sarkawt Hamad Ali, Omed Ikram Shihab

**Affiliations:** ^1^ The Department of Oral Diagnosis College of Dentistry Hawler Medical University, Erbil, Iraq; ^2^ The Department of Pedodontics Orthodontics and Preventive Dentistry College of Dentistry Hawler Medical University, Erbil, Iraq; ^3^ The Department of Oral and Maxillofacial Surgery College of Dentistry Hawler Medical University, Erbil, Iraq

## Abstract

**Objectives:**

This study aimed to evaluate and compare palatal thickness in adults for the placement of mini-implants for miniscrew-assisted rapid palatal expansion (MARPE) appliances using cone-beam computed tomography (CBCT) in a sample of Iraqi-Kurdish people.

**Materials and Methods:**

CBCT scans from 68 Kurdish patients, aged between 18 and 30 years, were assessed retrospectively. Of these, 37 were males and 31 were females. The measurements were performed at 3 mm from the mid-palatal suture. T-zone was selected for the anterior points, at the level of the palatal cusps of 2nd premolars, and the posterior point at the level of mesio-palatal cusps of 1st molars bilaterally. Palatal thickness of males and females bilaterally, as well as anterior and posterior areas, were measured and compared. An independent *t*-test was applied for comparison for normally distributed data, and the Mann–Whitney test was utilized for nonnormally distributed data. Additionally, Bonferroni correction was implemented for *p*-value adjustment.

**Results:**

The mean palatal thickness at the anterior area was 6.06 mm for males, 6.17 mm for females on the right side, 5.94 mm for males, and 5.99 mm for females on the left side. The mean palatal thickness at the posterior area was almost the same for both genders (4.40 mm for males and 4.44 mm for females) on the right side, 4.35 mm for males, and 4.54 mm for females on the left side. Statistically, no significant difference was recorded between males and females, as well as right and left sides in both anterior and posterior regions; however, a very highly statistically significant difference (*p*  < 0.001) was recorded when comparing total thickness, including both hard and soft tissue, between anterior and posterior regions.

**Conclusions:**

CBCT proves a highly effective modality in assessing palatal thickness and suggesting ideal locations for orthodontic mini-screw placement. Our examination of palatal thickness in a sample of Iraqi-Kurdish individuals revealed no statistical difference between genders or sides, but significant variations were noted between anterior and posterior thicknesses. Comprehensive clinical and pre-expansion CBCT evaluations are crucial for precisely determining the optimal placement of MARPE devices in each patient, ensuring successful outcomes.

## 1. Introduction

Transverse maxillary deficiency is a relatively common orthodontic problem that can present in one of three ways: bilateral posterior crossbite with teeth crowding, unilateral posterior crossbite with mandibular shifting, and constriction without crossbite. It can cause asymmetrical facial growth or retrusive mandible, temporomandibular joint disorder, and oral breathing [1].

Maxillary expansion is a predictable way to treatment of maxillary constriction and moderate maxillary crowding [2]. E. H. Angell' first reported on the procedure of maxillary expansion in 1860, and since then, it has gone through periods of popularity and decline [2].

The correction of the transverse discrepancy of the maxilla can be achieved with conventional rapid palatal expansion (RPE) by means of opening the mid-palatal suture, which is usually conducted in growing ages, whereas in nongrowing patients, where the increasing age is considered a limitation to achieving this transverse separation [3].

As a consequence, surgically assisted rapid maxillary expansion (SARME) was introduced to be used for mature patients by releasing the closed sutures resisting expansion force. It increases skeletal expansion efficiency and reduces the side effects, and it is necessary to adopt surgically assisted rapid palatal expansion (SARPE) instead [4]. However, SARME has higher biological and financial costs with most patients being reluctant to undergo surgical procedures [4].

Recently, fixed-type maxillary expansion appliances anchored to miniature implants have been developed as part of a technique known as miniscrew-assisted rapid palatal expansion (MARPE), which effectively has emerged as an alternative to SARPE [3]. In fact, miniscrews conventionally employed to avoid unwanted tooth movements can be used to obtain skeletal anchorage, as their mechanical properties allow sufficient resistance to orthopedic loads [5] if oriented perpendicularly to cortical bone forces [6]. This (MARPE) technique is indicated in skeletally mature patients to open the mid-palatal suture aiming at maximizing the skeletal effects because direct expansion forces are applied to the basal bone [7]. The MARPE appliance aimed to maximize the achievable expansion without enhancing the undesired inclination of the posterior teeth and collateral periodontal effects, thus allowing for stable posttreatment results. The survival rate of palatal mini-implants was generally high [8]. Hyperdivergent individuals typically exhibit a greater thickness of palatal bone in comparison to other groups [9].

The introduction of cone-beam computed tomography (CBCT) for use in the dental field in the late 1990s [10, 11, 12] and its proliferation over the last two decades can be considered a revolution in dental imaging [13]. Before the introduction of CBCT, “medical” computed tomography (CT) could be accessed, but with much higher radiation doses than those of traditional dental radiographs and CBCT [13]. Continuous development of system software for dental clinicians, in addition to the standard multiplanar reconstruction for orthogonal re-slicing (axial, coronal, and sagittal), adds to the potential diagnostic capabilities [13, 14]. Cross-sectional imaging modalities obviously permit more accurate localization of objects and remove superimpositions [15, 16].

A systematic review performed by De Vos et al. [17] showed that the clinical uses of CBCT included dento-alveolar and maxillofacial surgery, implantology, and specialized dentistry such as orthodontics [13]. CBCT is widely used by orthodontists in diagnosis, treatment planning, and evaluating treatment outcomes [18].

Various studies have attempted to evaluate the thickness of the palatal bone and its density to insert mini-implants. These investigations have been conducted on different populations and across various age groups [19, 20, 21, 22, 23]. In 2014, Nishii et al. [24] conducted a study to quantify palatal bone thickness in individuals from four distinct ethnic groups. Their findings revealed variations in palatal bone thickness across these ethnic groups, leading them to emphasize the need to consider ethnic diversity [24, 25].

In the literature, several MARPE designs [26, 27, 28] with different mini-implant lengths and insertion angles have been discussed, with reports emphasizing mainly the expansion results and lacking attention concerning how to conduct appropriate mini-implant selection with respect to the patient's anatomy. Thus, this study used CBCT images to evaluate and compare palatal thickness in adults at 3 mm from the mid-palatal suture for the placement of mini-implants for MARPE appliances.

## 2. Methods and Materials

This retrospective study is based on the assessment of CBCT scans obtained from the Department of Oral Diagnosis at the College of Dentistry—Hawler Medical University. These scans were justified and taken for different reasons following referral criteria.

The dental clinics at the College of Dentistry—Halwer Medical University operate as a public dental hospital. Patients receive notification that our clinic functions as a teaching facility. Their data may be utilized for research or teaching endeavors. Additionally, each department's case sheet includes a section, written in Kurdish—the local language—detailing these provisions.

For the selection of relevant CBCT datasets, the following inclusion criteria were adopted: participants aged between 18 and 30 years with no history of surgical treatment or craniofacial abnormalities, such as cleft lip and palate, bone disorders, or metabolic disorders. Images depicting the absence of maxillary first premolars or first molars, impacted canines, and supernumerary teeth were excluded.

The sample size was calculated using G^*∗*^Power (ver. 3.1.9.7) [29]. The power level has been set to 0.8 and the significance level to 5%. The results of the power calculation showed that a sample of 52 cases was necessary to obtain suitable data power. To exclude any risk of false-negative results, it was decided to increase the sample size to 68 cases, including 31 females and 37 males, from the 127 patient files that met the selection criteria.

### 2.1. CBCT Scanning

A Newtom Giano HR CBCT scanner (Quantitative Radiology/Cefla Dental Group/Imola BO/Italy) was used, operated with a full 360^o^ rotation and exposure parameters controlled by SafeBeam™ technology (“AEC” Automatic Exposure Control). Different fields of view were used, according to the CBCT scan requests, starting from 6 x 6 cm up to 10 × 8 cm, using a voxel size of 0.150 mm.

The CBCT image datasets were exported from the acquisition computer with the native viewing software (NewTom NNT™ software/Quantitative Radiology/Cefla Dental Group/Imola BO/Italy) to a Dell laptop (Inspiron 17 7000 Series 2-in-1—7773, Dell Inc., TX, USA) 17.3-inch FHD, IPS Truelife LED-backlit touch display with a screen resolution of 1,920 × 1,080 pixels, luminance of 330 cd/m^2^, and luminance contrast ratio of 1,538 : 1. The same laptop PC has been used in previous studies by the first author [13, 30, 31]. These parameters fulfilled the requirements for monitors used for clinical CBCT viewing [32].

### 2.2. CBCT Assessments

All CBCT scans were assessed by a single examiner (F.A.Y) Specialist in Dental and Maxillofacial Radiology who has more than 10 years of experience using (NewTom NNT™ software/Quantitative Radiology/Cefla Dental Group/Imola BO/Italy), to perform the necessary measurements. All assessments were made on the same laptop PC described above.

Fifteen CBCT datasets were randomly selected and remeasured after 3 weeks by the same examiner to permit the calculation of intra-observer repeatability.

**Figure 1:**
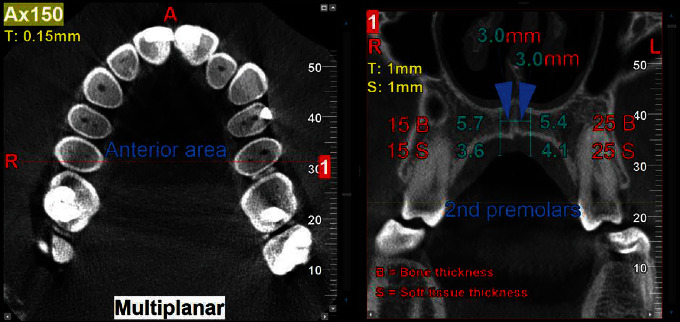
Axial cross-section showing the anterior line at the level of palatal cusps of 2nd premolars. Coronal cross-section showing the measurements of palatal thickness at the level of palatal cusps of 2nd premolars. Maxillary right 2nd premolar (15), maxillary left 2nd premolar (25).

**Figure 2:**
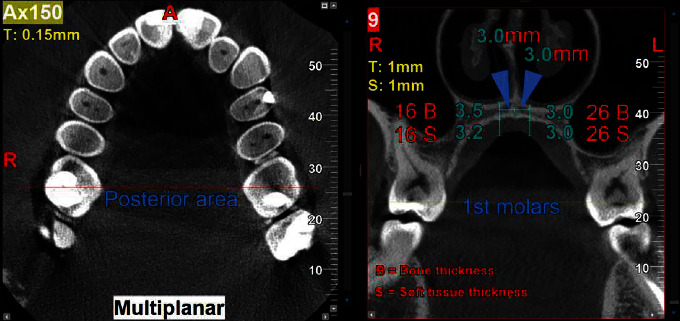
Axial cross-section showing the posterior line at the level of mesio-palatal cusp of 1st molars. Coronal cross-section showing the measurements of palatal thickness at the level of mesio-palatal cusp of 1st molars. Maxillary right 1st molar (16), maxillary left 1st molar (26).

The palatal thickness is measured on the multiplanar option of NNT software (version 14.0.1) as follows:

1. On the axial cross-section, a straight line was drawn anterio-posteriorly in T-zone, at the level connecting the palatal cusps of the 2nd premolar (distal to 3rd ruga area), which is the reference point for the anterior holes of the MARPE placement [33, 34, 35, 36, 37, 38, 39], so a series of multiple coronal sections produced at a setting of 1.0 mm slice thickness and 1.0 mm step interval between the slices (Figure 1).
2. The palatal thickness in image no. 1 and image no. 9 was measured as the distance between the anterior hole and posterior one of the MARPE device is 8.0 mm; thus, image no. 9 was at the level of mesio-palatal cusp of 1st molars bilaterally (Figure 2).
3. The right and left holes of MARPE are 6.0 mm apart, so two-line measurements were drawn at each level, 3.0 mm from the mid-palatal suture (Figures 1 and 2).
4. At the end of each line, i.e., 3.0 mm from the mid-palatal suture bilaterally, palatal thickness (hard and soft tissue) was measured as illustrated in Figures 1 and 2.

The thickness of hard and soft tissue was defined as the distance between the upper edge of the palatal bone (floor of nasal cavity) and the lower edge of palatal soft tissue (intraoral mucosa).

In this study, the thickness of palatal hard tissue and palatal soft tissue were measured. The thickness of palatal hard tissue was defined as the distance between the upper and lower edges of palatal bone (from the floor of the nasal cavity to the edge of palatal soft tissue), while the thickness of palatal soft tissue was measured as the distance between the lower edge of palatal bone and the lower edge of palatal soft tissue.

### 2.3. Statistical Analysis

All measurements were entered into a spreadsheet file (Excel, Microsoft 2016, Redmond, California). The statistical analysis, which encompassed both descriptive statistics and inferential statistics, was conducted using Statistical Package for the Social Sciences (SPSS) software (version 24.0; IBM Corporation, Armonk, NY). Intraexaminer reliability was evaluated using intraclass correlation coefficients (ICCs). The measurements exhibited a high level of reliability, as indicated by the ICC range of 0.76–0.89.

The normality of the data distribution was assessed using the Shapiro–Wilk test. A comparative analysis was conducted on the data obtained from both male and female subjects. Additionally, a comparison was made between the data collected from the right and left sides, as well as between the anterior and posterior data using an Independent *T*-test when the data exhibited normal distribution in both groups. However, when the data in one or both groups did not follow a normal distribution, the comparison was conducted using the Mann–Whitney test. The significance levels were established at a threshold of *p* < 0.05; however, due to the fact that multiple comparisons were conducted, the Bonferroni correction was applied, resulting in the adjustment of the *p*-value to 0.016.

The null hypothesis stated that there is no difference in palatal thickness between males and females, between the right and left sides, as well as anterior and posterior regions.

### 2.4. Ethical Aspects

The study protocol was reviewed and approved by Hawler Medical University Research Ethics Committee number (HMU.D.97).

## 3. Results

Descriptive statistics were computed for each of the investigated variables, specifically soft tissue thickness, bone thickness, and total thickness. The dataset comprises many statistical measures, such as the mean, confidence interval (95%), standard deviation, median, maximum, and minimum. The findings are displayed in Tables 1, 2, and 3.

The study involved measuring the mean values of total palatal thickness (bone and soft tissue) for mini-screw insertion at the upper right premolar location. The results indicated that males had a mean value of 6.06 mm, while females had a mean value of 6.17 mm. Comparably, it was observed that the average thickness in the upper left premolar region was 5.94 mm for males and 5.99 mm for females. Furthermore, it was observed that the overall thickness in the upper right molar was around 4.44 mm for both males and females. Specifically, the thickness was roughly 4.35 mm in males and 4.54 mm in females in the upper left molar region.

In this study, inferential statistics were utilized to examine and contrast data related to individuals of both male and female genders. The findings of this research have been provided in Table 4, revealing that there were no statistically significant disparities identified between the two genders when the total thickness, bone thickness, and soft tissue thickness were compared.

The research employed inferential statistics to examine the data obtained from the right and left sides (total thickness, bone thickness, and soft tissue thickness), as outlined in Table 5. The conducted research did not produce any statistically significant disparities between the two factions.

The data clearly indicate that the thickness of both the soft tissue and the bone of the palate is higher in the premolar region (anterior) compared to the molar region (posterior), varying from 6.11 mm to 4.42 mm, respectively. This observed difference was shown to be statistically very highly significant, with a *p*-value of less than 0.001, and it was observed in both males and females.

## 4. Discussion

The widespread adoption of the maxillary skeletal expander and its high success rate in expanding the constricted maxilla, particularly in adult patients, has had a transformative impact on the field. As a result, many orthodontists have sought to incorporate this appliance into their clinical practice. However, it is important to note that the success of MARPE is highly dependent on the specific techniques employed and necessitates a comprehensive understanding of the anatomical structure.

The achievement of successful screw engagement necessitates the involvement of both cortical layers. Therefore, in order to determine the optimal screw site and appropriate screw type and size, it is imperative to evaluate the thickness of the bone and soft tissue. The utilization of CBCT has the potential to enhance the effectiveness of this device prior to its placement.

This study is the initial endeavor to establish anatomical recommendations for the insertion of MARPE in the city of Erbil/Iraq, based on the current body of information.

The highly recommended MARPE appliance, due to its efficiency and high success rate, is very technique-sensitive, and many factors need to be fulfilled for its successful performance, like a good background in the anatomy of the palate and the knowledge of cortical bone thickness in different patients to address the best sites of insertion and the adequate implant size and length. Taking these points into consideration increases the chance of implant stability and reduces the risk of trauma to adjacent important tissues during insertion [40, 41, 42].

SARPE is another possible treatment approach for adult patients with a narrow maxilla, using bone cuts to reduce the resistance, followed by employing a jackscrew to expand and separate the two sides of the maxilla [43]. Unfortunately, this treatment is often rejected because it is an invasive surgical procedure with risks and high costs for the patient [44].

Radiological diagnosis prior to the placement of these implants is very helpful in planning the appliance placement with reduced failure risks, especially the use of CBCT evaluation before the insertion of implants, which increases the safety and the success rate of MARPE appliances [45, 46].

CBCT research that guides skeletal outcomes depending on clinical dental and soft tissue landmarks such as tooth cusps or Ruga areas are very helpful as a guideline for the clinicians during palatal implant insertion [7, 47, 48]. Other studies that provide clinically invisible landmarks are not useful as a guideline for the clinicians to help them with the insertion of these implants [49, 50, 51]. The visualization quality of the bony structures in CBCT appears to be similar to that offered by CT. However, CBCT generates high-resolution isotropic volume data and, therefore, could offer benefits in evaluating the maxilla thanks to the use of a lower radiation dose [12, 36, 37]. Thus, CBCT is a powerful modality for assessing palatal thickness and suggesting the perfect site for placement of orthodontic mini-screws [38, 39].

The constriction of the maxillary vault hinders the correct positioning of the expansion screw. Because the palate is shaped like a V, moving the screw more posteriorly will offer more palatal width [52, 53]; furthermore, the areas where resistance occurs during maxillary expansion are the mid-palatal suture, zygomatic buttress bone, and pterygopalatine suture [54]. To achieve maximum skeletal expansion, the force vector needs to be directed via the center of resistance, which is situated in the zygomatic buttress bone [55].

Previous studies in other ethnic groups proposed the third Ruga area as an indirect clinical landmark, comparing them to interdental areas [8]. This clinical landmark, which corresponds to the third Ruga area, is a stable clinical landmark, which is also proposed by other previous studies [3, 7]. To the best of our knowledge, this is the first study that investigates the palatal bone thickness for the insertion of MARPE appliance palatal mini-implants. It depends on the specific clinical landmark during palatal implant insertion in the Kurdish population. Therefore, the area distally to the 3rd Ruga, “i.e., at 2nd premolar level,” was chosen, as this is the safest area of T-zone implant placement in the Para median area covering the area of 1st and 2nd premolars bilaterally [35].

Considering the result of the current study, no statically significant differences were revealed considering the palatal bone thickness between males and females nor regarding the palatal soft tissue thickness, which is in agreement with the results of Becker et al. [56], who also found no significant difference in the palatal bone thickness between males and females. In contrast, other studies disagree with the results of the current study in that there was a significant difference in the palatal bone thickness between male and female patients [39, 49, 57], and this may be related to the difference in the ethnic group that is involved in the current study compared with the previous studies with different ethnicity.

Palatal bone thickness showed nonsignificant variability between the study participants, which is in disagreement with previous studies [15]. The mean values of palatal bone thickness in the current study revealed little variations, specifically varying from 4.08 to 4.15 mm for the left and right anterior regions, respectively. It shows 2.84–3.01 for the left and right posterior region, respectively, as well These values give the clinicians in our region an indication of the implant length to be selected for patients of Kurdish ethnicity; in addition, the thickness of soft tissue varied approximately between 2.11 mm for the anterior region and 1.65 mm for the posterior region, suggesting a selection of a miniscrew's neck of 2 mm to be enough. The value obtained from our study varies noticeably from other studies, being much less compared with previous studies, and this may be related to the sample size selected or the difference in the selected ethnic group [15].

Several factors can contribute to differences in palatal thickness between the right and left sides, including bone anatomy, tooth position, and individual variations in oral anatomy. In the current study, although there were discrepancies between the right and left sides, yet these variances were not statistically meaningful.

The thickness of the palatal bone is a significant determinant in the success of opening the mid-palatal suture with MARPE. As the thickness increases, the success rate also increases [58, 59]. Longer miniscrews, which can penetrate the bicortical bone (palatal and nasal cortical bone of the maxilla), have been recommended for greater orthopedic effects and parallel expansion at the coronal aspect [7]. The use of a long miniscrew for the posterior anchorage of MARPE increases the stability of the miniscrew. Nonetheless, longer miniscrews can damage the nasal floor mucosa or cause discomfort to the patient. MARPE with longer miniscrews does not guarantee midpalatal suture separation success, as per the data of the present study indicates that palatal thickness in the studied sample is somehow thin. Taking into account palatal shallowness variations and the available length of the miniscrew in the regional market varying between 10 and 16 mm. As the level of bone support has a pivotal role in determining the stability of miniscrews and enhancing the likelihood of successful MARPE treatment.

Likewise, the long-term durability of palatal mini-implants depends on the amount of palatal mucosa [60, 61]. Extremely thick palatal mucosa might increase the risk of inflammation [60, 61]. Thus, to minimize inflammation and maximize implant stability, the ideal insertion site for mini-implants is a location with robust palatal bone and reduced thickness of the linked palatal soft tissue. The current study indicates that a palatal mucosa thickness of 2.0 mm is recommended for effective adaption of the miniscrew neck.

Consistent with previous researches in the field, the findings of this study indicate a notable disparity in cortical bone and soft tissue thicknesses between the anterior and posterior regions. Specifically, as the measurements progressed from the anterior to posterior region toward the molar cusps area, a reduction in thickness was observed [39, 49, 57].

In the current study, palatal depth and cortical bone thickness were evaluated by insertion lines perpendicular to the palatal curvature plane, and this is in accordance with many other authors who applied this method [62, 63]. Others used perpendicular insert lines to the occlusal plane or to the constructed palatal planes, and some used angular insert lines [3, 7, 40, 42, 45, 64]. These methodological differences between studies involved in the measurement of palatal cortical bone thickness make the comparison somewhat difficult. In addition to the individual variations, therefore, the authors suggest that thorough clinical and pre-expansion CBCT examinations are essential for accurately determining the appropriate placement of the MARPE device in every patient to increase the success rate of these appliances.

Finally, although the sample of the current study includes adults rather than children, the majority of patients who undergo orthodontic therapy are children and young age [37, 65], which are radiosensitive and susceptible to improper effects of ionizing radiation; therefore, CBCT assessment should always be individually assessed and justified [66, 67].

### 4.1. Study Limitation

The study was a retrospective and observational study, with data obtained exclusively from one governorate teaching center. However, it should be noted that individuals from various areas attended this dental facility.

The aforementioned cases were referred to the Department of Oral Diagnosis/Dental X-Ray Section from other departments. Case notes in the X-ray section do not contain sufficient data to categorize them as either high-angle or low-angle faces.

## 5. Conclusion

CBCT is an overpowering modality for measuring palatal thickness and suggesting the perfect site for placement of orthodontic mini-screws individually. Our study of the palatal thickness on a sample of Iraqi-Kurdish people showed statistically no difference in the data between male and female, as well as the right and the left sides; however, there were significant differences between anterior and posterior palatal thicknesses. Thorough clinical and pre-expansion CBCT examinations are essential for accurately determining the appropriate placement of the MARPE device in every patient to guarantee the success.

## Figures and Tables

**Table 1:** Descriptive statistics of cortical thickness outcome and values are reported in millimeters (mm).

Tooth no.	Sex	Mean (95% CI)	Standard deviation	Median	Minimum	Maximum
Anterior area						
Upper right premolar region	M	4.15 (3.7–4.5)	1.27	4.2	2	7
	F	4.24 (3.6–4.8)	1.64	4.2	1.2	7.8
Upper left premolar region	M	4.08 (3.65–4.52)	1.3	4	1.2	7.2
	F	4.19 (3.66–4.72)	1.44	4	1.5	7.4
Posterior area						
Upper right 1st molar region	M	3.01 (2.61–3.42)	1.21	2.8	1.3	6.2
	F	3.15 (2.58–3.72)	1.55	2.9	1	7.8
Upper left 1st molar region	M	2.84 (2.43–3.25)	1.23	2.6	0.9	6.3
	F	3.27 (2.7–3.84)	1.54	2.9	1.1	7.2

**Table 2:** Descriptive statistics of mucosa thickness outcome and values are reported in millimeters (mm).

Tooth no.	Sex	Mean (95% CI)	Standard deviation	Median	Minimum	Maximum
Anterior area						
Upper right premolar region	M	2.11 (1.9–2.33)	0.64	2	1	4
	F	2.11 (1.84–2.38)	0.74	2	1.2	4.5
Upper left premolar region	M	2.11 (1.9–2.33)	0.64	2	1	4.3
	F	2.09 (1.8–2.38)	0.79	1.9	1	5.1
Posterior area						
Upper right 1st molar region	M	1.6 (1.47–1.73)	0.39	1.6	1	2.6
	F	1.56 (1.45–1.67)	0.3	1.5	1.1	2.1
Upper left 1st molar region	M	1.65 (1.51–1.79)	0.42	1.7	1	2.7
	F	1.54 (1.41–1.68)	0.37	1.4	1	2.4

**Table 3:** Descriptive statistics of cortical bone thickness and mucosa thickness outcome and values are reported in millimeters (mm).

Tooth no.	Sex	Mean (95% CI)	Standard deviation	Median	Minimum	Maximum
Anterior area						
Upper right premolar region	M	6.06 (5.58–6.54)	1.44	6.1	3.9	9.1
	F	6.17 (5.7–6.64)	1.28	5.8	3.8	8.7
Upper left premolar region	M	5.94 (5.54–6.42)	1.44	5.9	2.7	8.4
	F	5.99 (5.52–6.46)	1.28	5.9	3.2	8.6
Posterior area						
Upper right 1st molar region	M	4.4 (4.02–4.79)	1.15	4.3	2.3	6.5
	F	4.44 (3.99–4.9)	1.23	4.3	2.6	7
Upper left 1st molar region	M	4.35 (4.01–4.7)	1.03	4.4	2.4	6.6
	F	4.54 (4.1–4.99)	1.21	4.5	2.6	6.8

**Table 4:** Inferential statistics performed to detect any significant difference between males and females.

	Anterior area		Posterior area	
	Right 2nd premolar area	Left 2nd premolar area	Right 1st molar area	Left 1st molar area
Mucosa				
Shapiro–Wilk test				
Male	*p*=0.31	*p*=0.029	*p*=0.069	*p*=0.151
Female	*p*=0.007	*p* < 0.001	*p*=0.115	*p*=0.031
Comparison test	*p*=0.772( ^*∗∗*^)	*p*=0.5( ^*∗∗*^)	*p*=0.709( ^*∗*^)	*p*=0.319( ^*∗∗*^)
Cortical bone				
Shapiro–Wilk test				
Male	*p*=0.65	*p*=0.433	*p*=0.007	*p*=0.001
Female	*p*=0.597	*p*=0.631	*p*=0.029	*p*=0.075
Comparison test	*p*=0.756( ^*∗*^)	*p*=0.797( ^*∗*^)	*p*=0.956( ^*∗∗*^)	*p*=0.254( ^*∗∗*^)
Total				
Shapiro–Wilk test				
Male	*p*=0.102	*p*=0.437	*p*=0.461	*p*=0.477
Female	*p*=0.271	*p*=0.818	*p*=0.129	*p*=0.19
Comparison test	*p*=0.752( ^*∗*^)	*p*=0.875( ^*∗*^)	*p*=0.883( ^*∗*^)	*p*=0.498( ^*∗*^)

^*∗*^*T* test (parametric data).  ^*∗∗*^Mann–Whitny *U* test (nonparametric data).

**Table 5:** Inferential statistics performed to detect any significant difference between the right and left sides in males and females.

	Mucosa		Cortical bone		Total	
	2nd premolar area	1st molar area	2nd premolar area	1st molar area	2nd premolar area	1st molar area
Male						
Shapiro–Wilk test						
Right	*p*=0.31	*p*=0.069	*p*=0.65	*p*=0.007	*p*=0.102	*p*=0.461
Left	*p*=0.029	*p*=0.151	*p*=0.433	*p*=0.001	*p*=0.437	*p*=0.477
Comparison test	*p*=0.811( ^*∗∗*^)	*p*=0.589( ^*∗*^)	*p*=0.816( ^*∗*^)	*p*=0.482( ^*∗∗*^)	*p*=0.712( ^*∗*^)	*p*=0.858( ^*∗*^)
Female						
Shapiro–Wilk						
Right	*p*=0.007	*p*=0.115	*p*=0.597	*p*=0.029	*p*=0.271	*p*=0.129
Left	*p* < 0.001	*p*=0.031	*p*=0.631	*p*=0.075	*p*=0.818	*p*=0.19
Comparison test	*p*=0.751( ^*∗∗*^)	*p*=0.57( ^*∗∗*^)	*p*=0.883( ^*∗*^)	*p*=0.783( ^*∗∗*^)	*p*=0.588( ^*∗*^)	*p*=0.757( ^*∗*^)

^*∗*^*T* test (parametric data).  ^*∗∗*^Mann–Whitney *U* test (nonparametric data).

## Data Availability

The data that support the findings of this study are available on request from the corresponding author. The data are not publicly available due to privacy or ethical restrictions.
